# Ivabradine Use in Premature Infants With Accelerated Idioventricular Rhythm

**DOI:** 10.1111/jce.16755

**Published:** 2025-07-09

**Authors:** Nawin L. Ramdat Misier, Pezad N. Doctor, Kaitlin Tindel, Natasja M. S. de Groot, Jordan E. Ezekian, Hoang H. Nguyen

**Affiliations:** ^1^ Department of Pediatrics The University of Texas Southwestern Medical Center Dallas Texas USA; ^2^ Department of Cardiology Erasmus Medical Center Rotterdam The Netherlands; ^3^ Department of Pharmacy Dallas Children's Medical Center Dallas Texas USA

**Keywords:** accelerated Idioventricular rhythm, arrhythmias, congenital heart disease, ivabradine, prematurity, ventricular tachycardia

## Abstract

**Introduction:**

Ivabradine has been recently introduced to treat focal atrial tachycardia and junctional ectopic tachycardia in children. Its use for treating ventricular tachyarrhythmias, specifically accelerated idioventricular rhythm (AIVR), remains limited.

**Methods and Results:**

We report two cases of premature infants with AIVR successfully treated with ivabradine as monotherapy after other antiarrhythmic agents had failed. The first infant had a structurally normal heart and the second infant a truncus arteriosus. In both patients, sinus rhythm was restored with ivabradine. Side effects only consisted of brief, hemodynamically insignificant episodes of bradycardia.

**Conclusions:**

Ivabradine represents a promising antiarrhythmic agent for treating AIVR in preterm infants. Its ability to maintain hemodynamic stability in this age group makes ivabradine an attractive option for managing AIVR, especially in the postoperative setting. Further research is warranted to explore the safety, dosage, and mechanistic action of ivabradine for AIVR in the premature, neonatal infant patient population.

**Abbreviations**
AIVR: accelerated idioventricular rhythm
HCN: hyperpolarization‐activated, cyclic nucleotide‐gated
NEC: necrotizing enterocolitis

## 1 Introduction

Accelerated idioventricular rhythm (AIVR) is a rare ventricular tachyarrhythmia observed in neonates. Although AIVR is generally benign and self‐limiting, it can occasionally lead to significant hemodynamic instability, necessitating intervention, especially in vulnerable premature infants and those with congenital heart disease [1, 2, 3, 4, 5]. Traditional antiarrhythmic drugs, including beta‐blockers, amiodarone, and sotalol, have been used to manage AIVR with variable success [3, 4]. However, their use can be complicated by side effects such as hypotension and bradycardia.

Ivabradine is a selective inhibitor of hyperpolarization‐activated cyclic nucleotide‐gated (HCN) channels, which decreases automaticity in the sinoatrial and atrioventricular nodes without affecting myocardial contractility or causing significant hypotension [6]. Ivabradine has been successfully used to treat inappropriate sinus tachycardia and sinus tachycardia associated with heart failure in children and adults [7]. Recently, it has been utilized to treat other pediatric tachyarrhythmias with an enhanced automaticity mechanism, such as junctional ectopic tachycardia and focal atrial tachycardia [8, 9, 10].

In this report, we describe two premature infants with AIVR who were successfully treated with ivabradine as monotherapy after other antiarrhythmic agents had failed. These cases demonstrate ivabradine's potential to treat ventricular tachyarrhythmias in the premature neonatal patient population.

## 2 Case Descriptions

### 2.1 Case 1

A 37‐day‐old male infant, born at 25 weeks of gestation, presented with incessant and hemodynamically stable wide complex tachycardia (160–180 beats per minute) on telemetry. The baseline sinus heart rate was 140–150 beats per minute. The pregnancy was complicated by recurrent vaginal bleeding associated with a lower uterine segment hematoma and an irregular fetal heart rhythm observed during a routine visit. However, a fetal echocardiogram showed normal cardiac anatomy and a regular rate and rhythm with 1:1 AV conduction, and the irregular heart rhythm did not recur. A cesarean section was performed because of decreased fetal tones (APGAR score 2‐7‐7).

**Figure 1:**
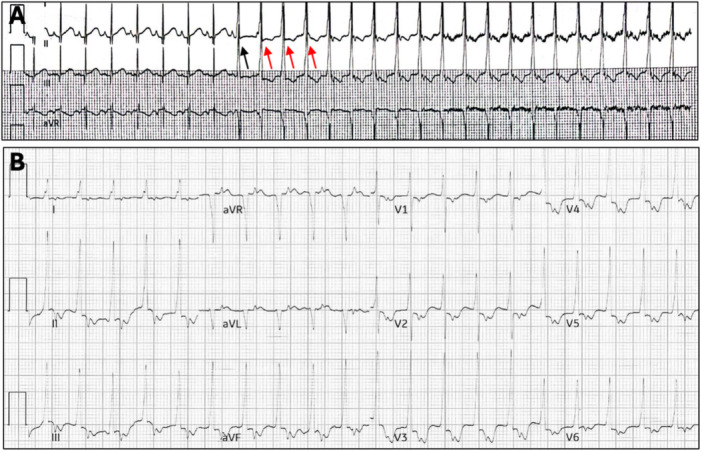
Patient 1. (A) Initiation of accelerated idioventricular rhythm at a rate slightly faster than the sinus rhythm rate with first being a fusion beat (black arrow) followed by a wide QRS complex (80 ms). Retrograde P waves noted with ventriculo‐atrial time of 110 ms after three initial QRS beats suggesting retrograde entrainment of atrium, indicated by the red arrows. (B) 12 lead‐electrocardiogram showing wide complex tachycardia with retrograde p waves following QRS complex (90 ms) suggestive of accelerated idioventricular rhythm. The AIVR was localized to the posterior right ventricular outflow tract.

The neonate's electrocardiogram revealed a regular wide QRS tachycardia, normal frontal QRS axis, and a lack of concordance across the precordial leads, as shown in Figure 1. There was 1:1 retrograde ventriculo‐atrial conduction. Except for a large persistent ductus arteriosus, the patient's transthoracic echocardiogram and blood electrolytes were normal. Adenosine (0.2 mg/kg) successfully terminated the tachycardia. However, the tachycardia reinitiated with a fusion beat at a rate slightly faster than the sinus rate, leading to a diagnosis AIVR (Figure 1A), which was localized to the posterior right ventricular outflow tract (Figure 1B) [11].

Given the infant's prematurity and the high burden of AIVR, enteral propranolol was initiated at 2 mg/kg/day, resulting in conversion to sinus rhythm after the first dose. Unfortunately, AIVR recurred 12 h later and persisted despite escalating the propranolol dose to 4 mg/kg/day. Ivabradine (0.1 mg/kg enterally) was then administered, successfully converting AIVR to sinus rhythm within 1 h after administration of the first dose. The patient was subsequently maintained on ivabradine at 0.05 mg/kg/dose enterally twice daily.

Propranolol was discontinued after ivabradine achieved steady state level. After administration of ivabradine for 3 days, the patient developed short, self‐resolving episodes of sinus bradycardia. The ivabradine dose was reduced to 0.03 mg/kg/dose twice daily. After two reduced doses, the heart rate returned to sinus rhythm. AIVR, however, recurred on the same day, prompting an increase in the ivabradine dose back to 0.05 mg/kg/dose twice daily, with conversion to sinus rhythm after the first increased dose. Bradycardia was not further observed during ivabradine therapy.

Three weeks following ivabradine initiation, he had an episode of pneumatosis which prompted concerns for necrotizing enterocolitis (NEC). Subsequently, all enteral medications were stopped. AIVR recurred 48 h after ivabradine was discontinued. The patient remained hemodynamically stable and given the patient's older age and thus presumed increased hemodynamic tolerance for the arrhythmia, it was decided not to start intravenous antiarrhythmic medication while the patient was NPO. The patient was treated with antibiotics for 7‐days. In the following 2 weeks, the frequency of AIVR gradually decreased, and the patient eventually returned to a permanent sinus rhythm. AIVR has not recurred at 10 months of age. The patient did experience another episode of pneumatosis and emesis 3 weeks after stopping ivabradine, for which he was treated with antibiotics for 3 days.

### 2.2 Case 2

**Figure 2:**
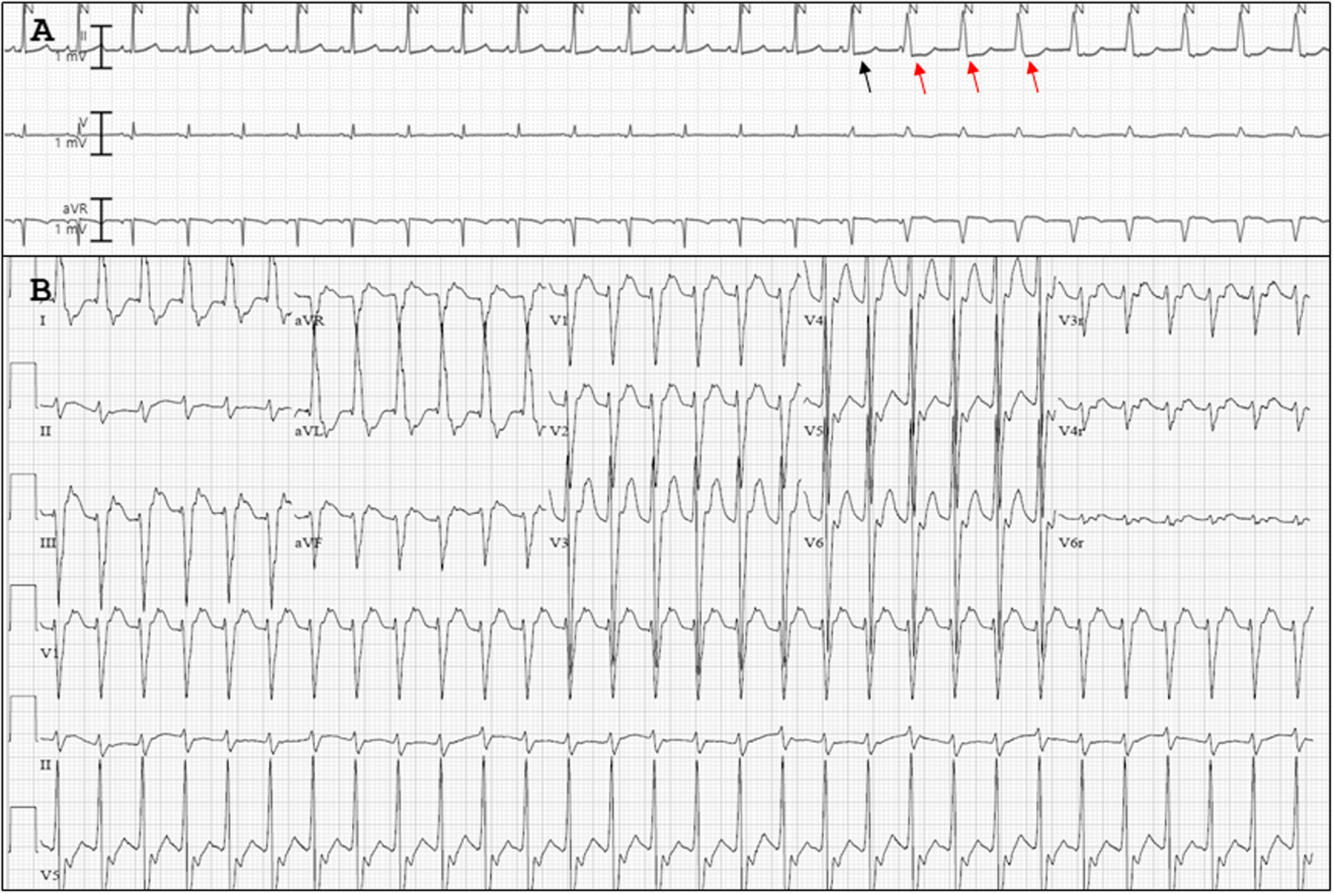
Patient 2. (A) Initiation of accelerated idioventricular rhythm at a rate slightly faster than the sinus rhythm rate with first being a fusion beat (black arrow) followed by a wide QRS complex (110 ms). Retrograde P waves with ventriculo‐atrial conduction time of 115 ms after three initial QRS beats suggest retrograde entrainment of atrium, indicated by the red arrows. (B) 12 lead‐electrocardiogram showing wide complex tachycardia with retrograde p‐waves following QRS complex suggestive of accelerated idioventricular rhythm. Due to the uncorrected truncus arteriosus, localization of the AIVR with conventional PVC‐origin algorithm was deemed to be suboptimal and inaccurate.

A 6‐day‐old male infant, born at 34 weeks of gestation with truncus arteriosus, underwent bilateral pulmonary artery banding. Eight hours postoperatively, he developed incessant wide complex tachycardia (130–180 beats per minute), as shown in Figure 2B. The diagnosis of AIVR was made based on the tachycardia rate being within 20% of the sinus rate, AIVR initiation with a fusion beat, and the presence of ventriculo‐atrial dissociation.

Initially, he remained hemodynamically stable during the AIVR; however, after 6 h, he became hemodynamically unstable reflected by poor systemic perfusion, widening of the arterio‐venous oxygen saturation difference, and rising lactate levels. Despite these changes, his echocardiogram showed normal biventricular systolic function. A loading dose of lidocaine (1 mg/kg) was given, followed by a continuous infusion (30 mcg/kg/min). However, lidocaine was discontinued after 8 h as AIVR persisted. Subsequently, an esmolol infusion was started at 25 mcg/kg/min and titrated up to 50 mcg/kg/min, but it failed to restore sinus rhythm and caused hypotension, resulting in discontinuation of esmolol.

Ivabradine (0.1 mg/kg) was administered enterally, and conversion to sinus rhythm occurred within 1 h of the first dose. The patient received a maintenance dose of ivabradine at 0.05 mg/kg/dose enterally twice daily. Once in sinus rhythm, his hemodynamic markers improved, allowing for weaning of inotropic drugs and extubation within 24 h after ivabradine initiation. While using ivabradine, he remained in sinus rhythm.

After 13 days of maintenance ivabradine therapy, the patient developed NEC. Abdominal aortic echography demonstrated (trivial) reversal of diastolic flow, which could contribute to the development of NEC through mesenteric circulatory insufficiency and is related to truncus arteriosus. The NEC required surgical management and he was not fed enterally but continued receiving ivabradine. After 18 days of maintenance ivabradine therapy, the patient developed bradycardia; his average heart rate decreased from 120 to 100 beats per minute. Although this was hemodynamically well tolerated, the ivabradine dose was reduced to 0.025 mg/kg/dose twice daily, resulting in increase in heart rate to 120 beats per minute after two reduced doses. Ivabradine was subsequently discontinued after 22 days. He remains in sinus rhythm without recurrence of AIVR at the age of 8 months, and has since undergone complete surgical repair of his truncus arteriosus.

## 3 Discussion

AIVR is typically considered benign and often does not require treatment in infants. However, in specific cases, particularly those involving congenital heart disease and/or preterm neonates, hemodynamic compromise can arise, as demonstrated in our second case. In our first case, despite the absence of immediate hemodynamic instability, the infant was treated proactively. This decision was driven by the significant burden of AIVR, the infant's extreme prematurity and associated challenges.

There is no standardized protocol for selecting antiarrhythmic agents to manage AIVR in neonates after failed beta‐blocker treatment, and treatments have yielded inconsistent results [12]. In our experience, conventional treatments with propranolol, lidocaine, and esmolol often fail to achieve rhythm conversion. While there is a paucity of data on the use of ivabradine in treatment of AIVR in (preterm) infants, ivabradine has proven to be safe for use in infants for atrial tachyarrhythmias [7, 10]. Therefore, based on the previous experience with ivabradine therapy for atrial tachyarrhythmias, an empiric approach for using ivabradine to treat neonatal AIVR was taken.

In our two cases, ivabradine proved to be effective, converting both infants to sinus rhythm after a single 0.1 mg/kg dose. The temporal relationship between the onset of AIVR and the decrease in dose and discontinuation of ivabradine in the first case suggests that AIVR is responsive to ivabradine. In the second case, ivabradine achieved an acute termination of AIVR, and the rhythm remained stable during maintenance therapy. The absence of AIVR recurrences upon discontinuation of ivabradine in the second case demonstrates that the arrhythmia was limited to the immediate postoperative period.

It is assumed that AIVR results primarily from enhanced automaticity in the His‐Purkinje system or contractile ventricular myocytes [13]. Overexpression of HCN channels, which ivabradine directly inhibits, may contribute to this abnormal automaticity. A preclinical study in rabbits with idiopathic ventricular tachycardia indeed suggests that Purkinje fibers exist in the right ventricular outflow tract with overexpression of HCN channels [14]. A less common mechanism underlying AIVR is triggered activity, which was likely the underlying mechanism in the first case, where AIVR responded to adenosine and was caused by an ectopic focus in the right ventricular outflow tract [15]. Triggered activity, which can be terminated with adenosine, results from catecholamine‐induced cAMP‐mediated delayed after‐depolarizations. A preclinical study in rats with nonreperfused myocardial infarction suggests that ivabradine reduces the sensitivity of ryanodine receptors, potentially addressing the pro‐arrhythmic calcium leaks associated with triggered activity [16]. However, further investigation is needed to fully understand ivabradine's antiarrhythmic mechanism in neonatal AIVR.

Throughout the course of maintenance dosing, both patients developed hemodynamically insignificant bradycardia. Bradycardia developed at 3 days of maintenance ivabradine in the first patient and at 3 weeks in the second patient. While both patients' heart rates increased with a reduced dose of ivabradine, bradycardia also did not recur once the higher dose was re‐established in the first patient. Previous reports on ivabradine use in children with focal atrial tachycardia showed nonhemodynamically significant bradycardia and functional bradycardia with blocked premature atrial contractions [10]. Additionally, there are no appreciable descriptions to date of pharmacokinetic and pharmacodynamic response to ivabradine in young infants and neonates, especially in patients of substantial prematurity. The dosing for the AIVR indication was extrapolated from robust cardiomyopathy trials and proved to yield similar clinical response to study participants [7]. While further studies are needed to elucidate optimal dosing strategies for these special populations, our two cases provide an important first step.

While both cases developed NEC, we do not believe that this finding was directly associated with ivabradine. At present, there is no evidence of increased NEC development in neonates treated with ivabradine. Also, NEC is common in preterm infants, particularly in those with persistent ductus arteriosus and truncus arteriosus, which is in part related to mesenteric insufficiency by reversal of diastolic flow [17]. Specifically, the first patient had recurrent concerns for NEC while not on ivabradine and in presence of a persistent ductus arteriosus, while the second patient had a truncus arteriosus and demonstrated reversal of diastolic aortic flow.

## 4 Conclusion

Ivabradine represents a promising antiarrhythmic agent for treating AIVR in preterm infants. Its ability to maintain hemodynamic stability in this age group makes ivabradine an attractive option for managing AIVR, especially in the postoperative setting. Future studies are critical to further define the optimal dosage, safety profile, and efficacy of ivabradine for treatment of ventricular arrhythmias, ensuring its broader and safer application in the preterm, neonatal population.

## Conflicts of Interest

The authors declare no conflicts of interest.

## Data Availability

The data that support the findings of this study are available from the corresponding author upon reasonable request.
